# Supplementary material for: Chronic pain precedes disrupted eating behavior in low-back pain patients
Source: PLoS One. 2022 Feb 10;17(2):e0263527. doi: 10.1371/journal.pone.0263527 (PMC8830732; doi:10.1371/journal.pone.0263527)
Supplement: S1 Fig — A higher proportion of CLBP subjects reported problems with food high in sugar or fat (eg. ice cream, p = 0.04; cookies, p = 0.02; hamburger, p = 0.02; rice, and p = 0.02; Pearson Chi-square test) than HC. The proportion of participants reporting no problem with different food items was significantly higher in HC than CLBP. The proportion of SBP patients was somewhere in the middle for several items (e.g. ice cream, chocolate). Abbreviations: CLBP, chronic low back pain; SBP, subacute back pain; HC, Healthy Control; YFAS, Yale Food Addiction Scale. (DOCX) [file pone.0263527.s001.docx]

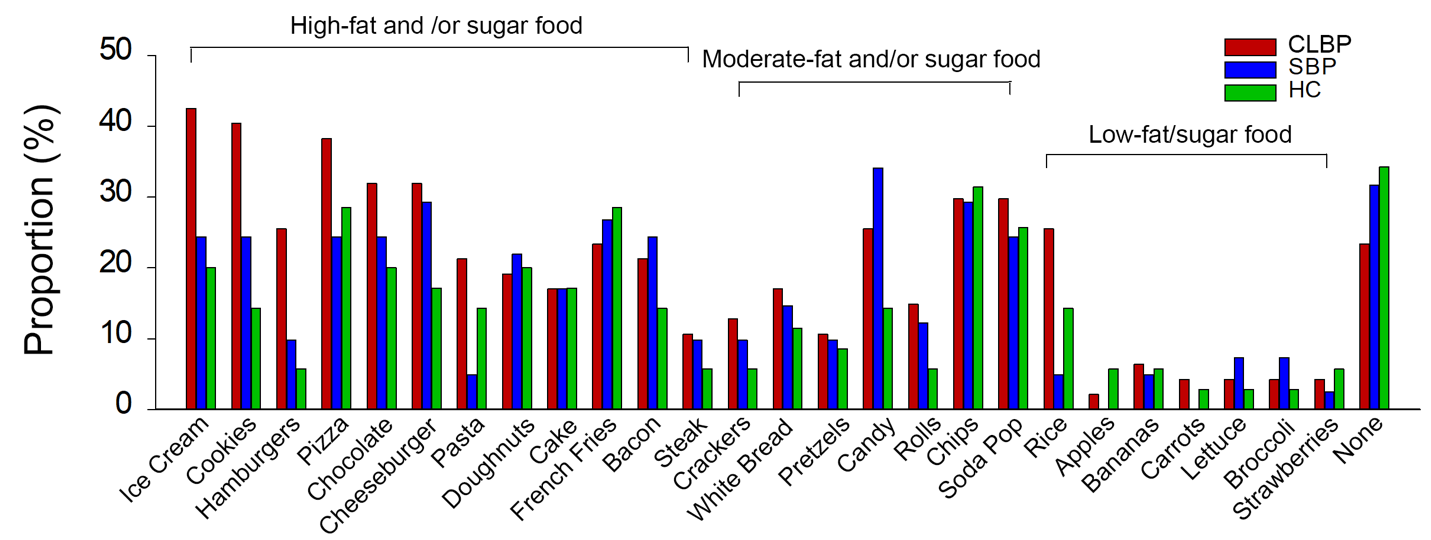


**S1Fig.** Percentage of participants from each group reporting “problems” with dietary items listed in YFAS. A higher proportion of CLBP subjects reported problems with food high in sugar or fat (eg. ice cream, p = 0.04; cookies, p = 0.02; hamburger, p = 0.02; rice, and p = 0.02; Pearson Chi-square test) than HC. The proportion of participants reporting no problem with different food items was significantly higher in HC than CLBP. The proportion of SBP patients was somewhere in the middle for several items (e.g. ice cream, chocolate). *Abbreviations:* *CLBP, chronic low back pain; SBP, subacute back pain; HC, Healthy Control; YFAS, Yale Food Addiction Scale.*
