# Supplementary material for: Chronic pain precedes disrupted eating behavior in low-back pain patients
Source: PLoS One. 2022 Feb 10;17(2):e0263527. doi: 10.1371/journal.pone.0263527 (PMC8830732; doi:10.1371/journal.pone.0263527)
Supplement: S2 Fig — SBPr, subacute back pain recovered; SBPp, subacute back pain persistent; HC, Healthy Control; YFAS, Yale Food Addiction Scale. (DOCX) [file pone.0263527.s002.docx]

**
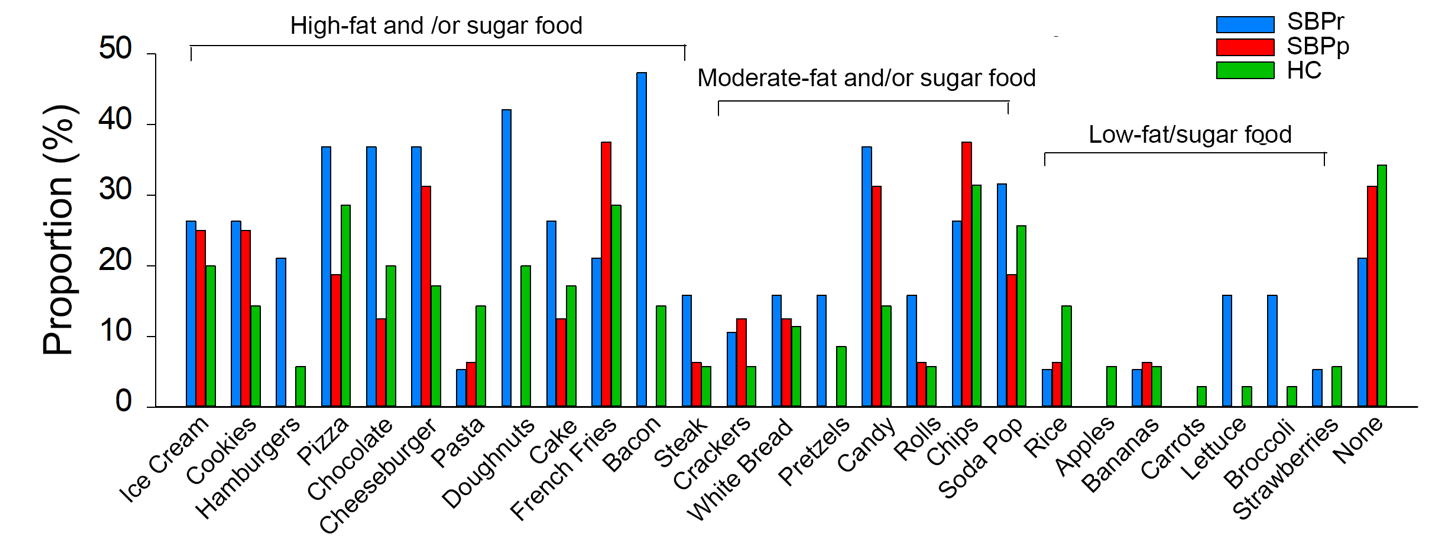
S2 Fig**. Problematic food listed in YFAS for SBP groups and HC at baseline. SBPr, subacute back pain recovered; SBPp, subacute back pain persistent; HC, Healthy Control; YFAS, Yale Food Addiction Scale.
