# Supplementary figures and images for: Chronic pain precedes disrupted eating behavior in low-back pain patients
Source: PLoS One. 2022 Feb 10;17(2):e0263527. doi: 10.1371/journal.pone.0263527 (PMC8830732; doi:10.1371/journal.pone.0263527)

**
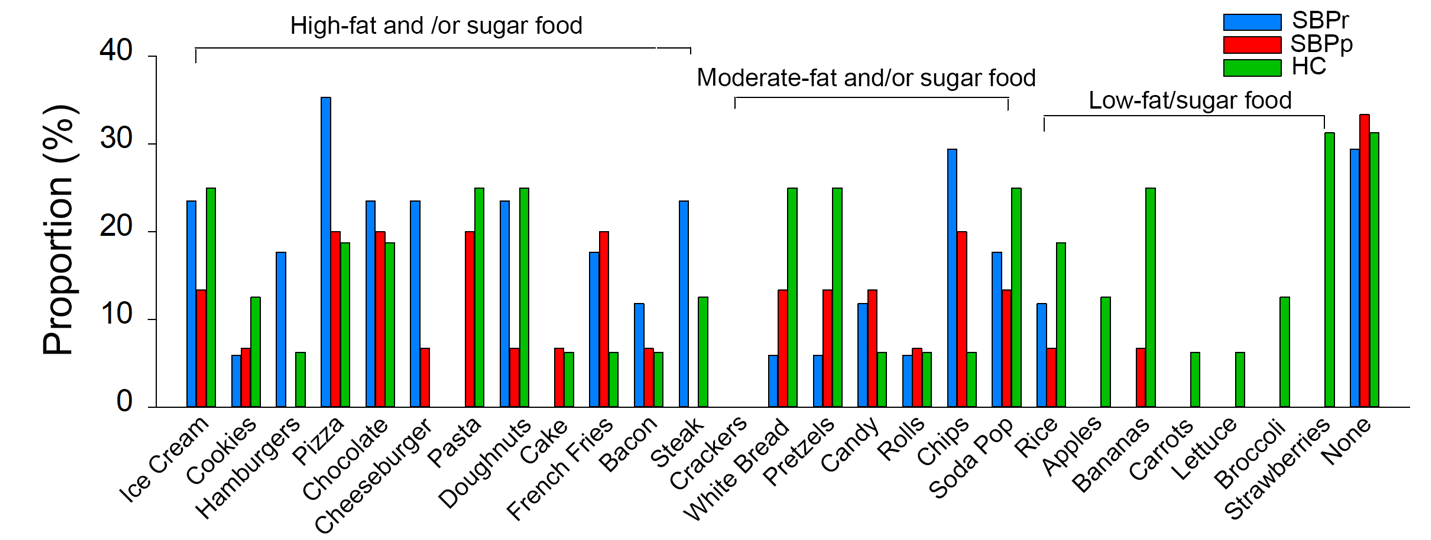
**

**S3 Fig**. Problematic food listed in YFAS for SBP groups and HC at follow-

up.

Supplement: S3 Fig — (DOCX) [file pone.0263527.s003.docx]
