# Supplementary material for: Chronic pain precedes disrupted eating behavior in low-back pain patients
Source: PLoS One. 2022 Feb 10;17(2):e0263527. doi: 10.1371/journal.pone.0263527 (PMC8830732; doi:10.1371/journal.pone.0263527)
Supplement: S4 Fig — (A) Hunger rated by SBP, CLBP and HC participants; (B) Fullness; (C) Thirst. (DOCX) [file pone.0263527.s004.docx]

**
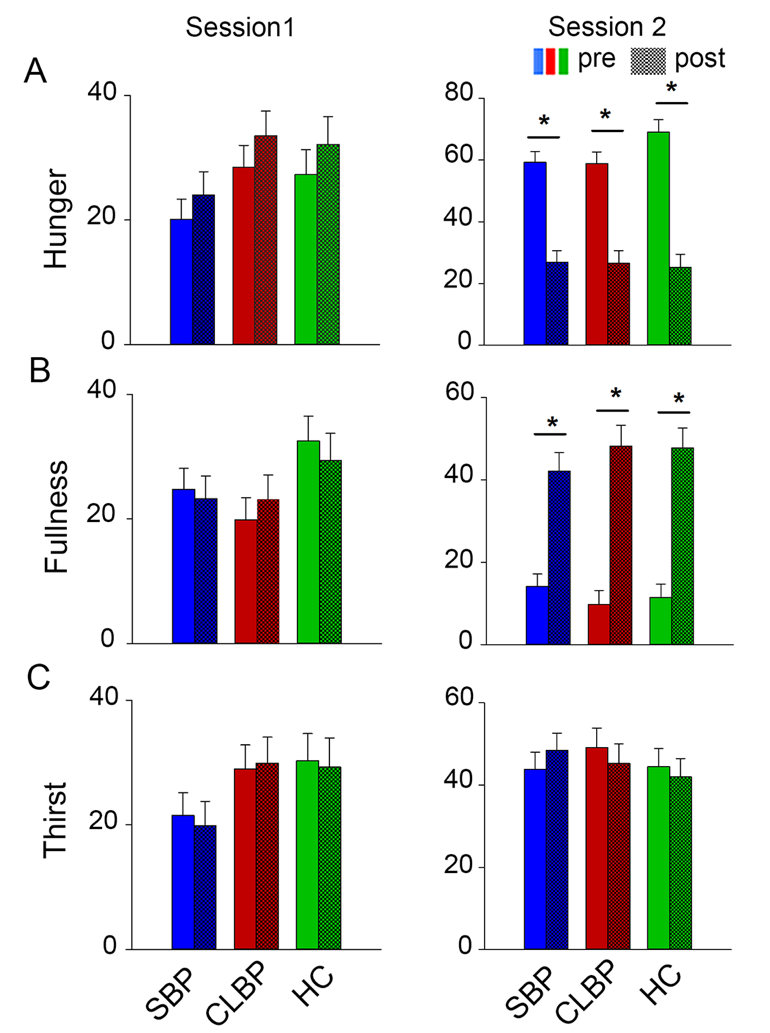
**

**S4 Fig**. Internal ratings of SBP, CLBP and HC subjects during session 1 (left) and 2 (right). (**A**) Hunger rated by SBP, CLBP and HC participants; (**B**) Fullness; (**C**) Thirst.
