# Supplementary material for: Chronic pain precedes disrupted eating behavior in low-back pain patients
Source: PLoS One. 2022 Feb 10;17(2):e0263527. doi: 10.1371/journal.pone.0263527 (PMC8830732; doi:10.1371/journal.pone.0263527)
Supplement: S5 Fig — A for SBP (n = 51), CLBP (n = 43 for pudding and n = 41 for Jello) and healthy control subjects (n = 36) at baseline; B for SBPr (n = 20), SBPp (n = 16) and HC (n = 36) at baseline and C for SBPr (n = 15 for pudding and n = 13 for Jello), SBPp (n = 13) and HC (n = 16 for pudding and n = 14 for Jello) at follow-up. (A–C) (Left) Participants’ ratings for each of the 4 fat concentrations of puddings. (Middle) Ratings for each of the 4 sucrose concentrations of orange-flavored Jello. (Far right) Bar plot depicting mean ± standard error of the mean (SEM) across all the concentrations for pudding (left) and juice (right). No group effect, concentration effect or interaction was found. Adding covariates, including pretest hunger rating and sex did not affect the main results. (DOCX) [file pone.0263527.s005.docx]

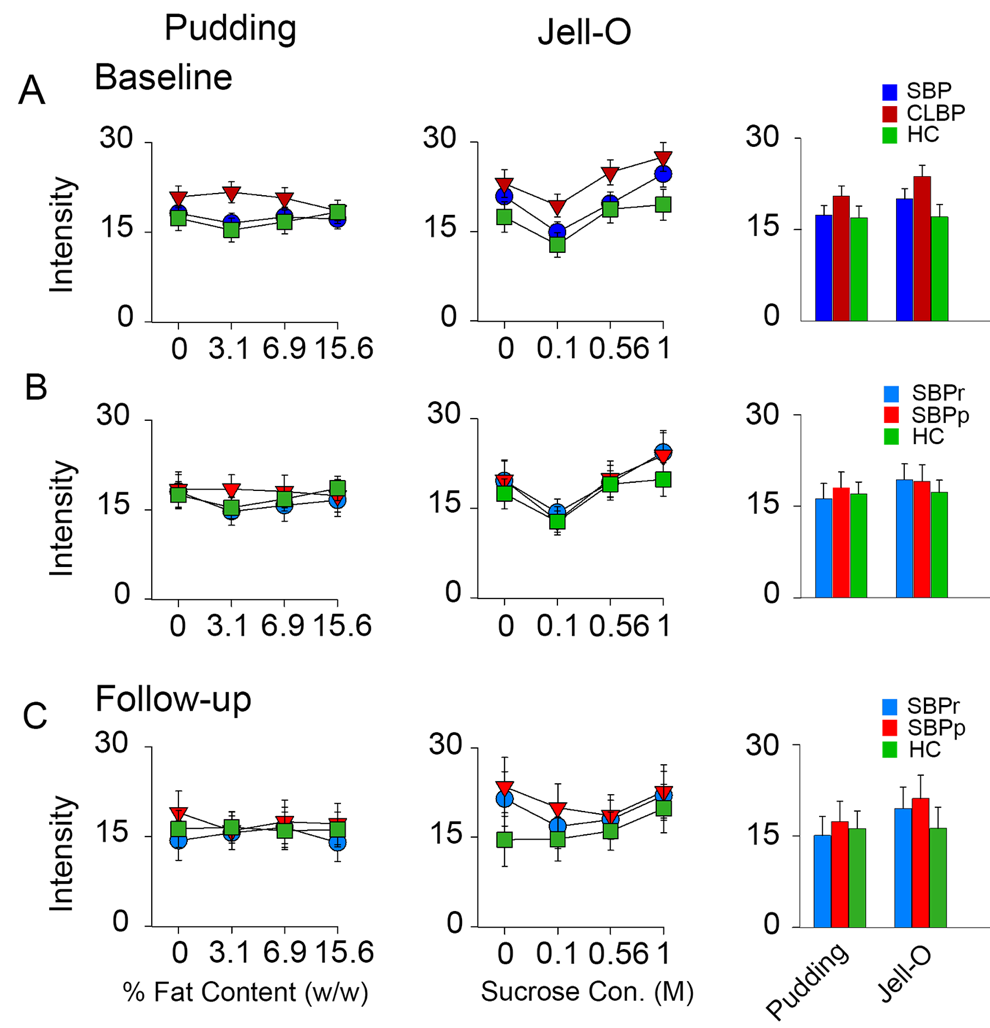


**S5 Fig**. Psychophysical ratings (intensity) during session 1 at baseline and follow-up. A for SBP (n = 51), CLBP (n = 43 for pudding and n = 41 for Jello) and healthy control subjects (n = 36) at baseline; B for SBPr (n = 20), SBPp (n = 16) and HC (n = 36) at baseline and C for SBPr (n = 15 for pudding and n = 13 for Jello), SBPp (n = 13) and HC (n = 16 for pudding and n = 14 for Jello) at follow-up. (A–C) (Left) Participants’ ratings for each of the 4 fat concentrations of puddings. (Middle) Ratings for each of the 4 sucrose concentrations of orange-flavored Jello. (Far right) Bar plot depicting mean ± standard error of the mean (SEM) across all the concentrations for pudding (left) and juice (right). No group effect, concentration effect or interaction was found. Adding covariates, including pretest hunger rating and sex did not affect the main results.
