# Supplementary material for: Chronic pain precedes disrupted eating behavior in low-back pain patients
Source: PLoS One. 2022 Feb 10;17(2):e0263527. doi: 10.1371/journal.pone.0263527 (PMC8830732; doi:10.1371/journal.pone.0263527)
Supplement: S6 Fig — (A) Ratings of MacCheese (left) and pudding (right) for SBP, CLBP and HC participants at baseline; (B) Ratings of MacCheese (left) and pudding (right) for SBPr, SBPp and HC participants at baseline; (C) Ratings of MacCheese (left) and pudding (right) for SBPr, SBPp and HC at follow-up. CLBP, chronic low back pain; SBP, subacute back pain; SBPr, subacute back pain recovered; SBPp, subacute back pain persistent; HC, Healthy Control. No significant differences were found among groups. (DOCX) [file pone.0263527.s006.docx]

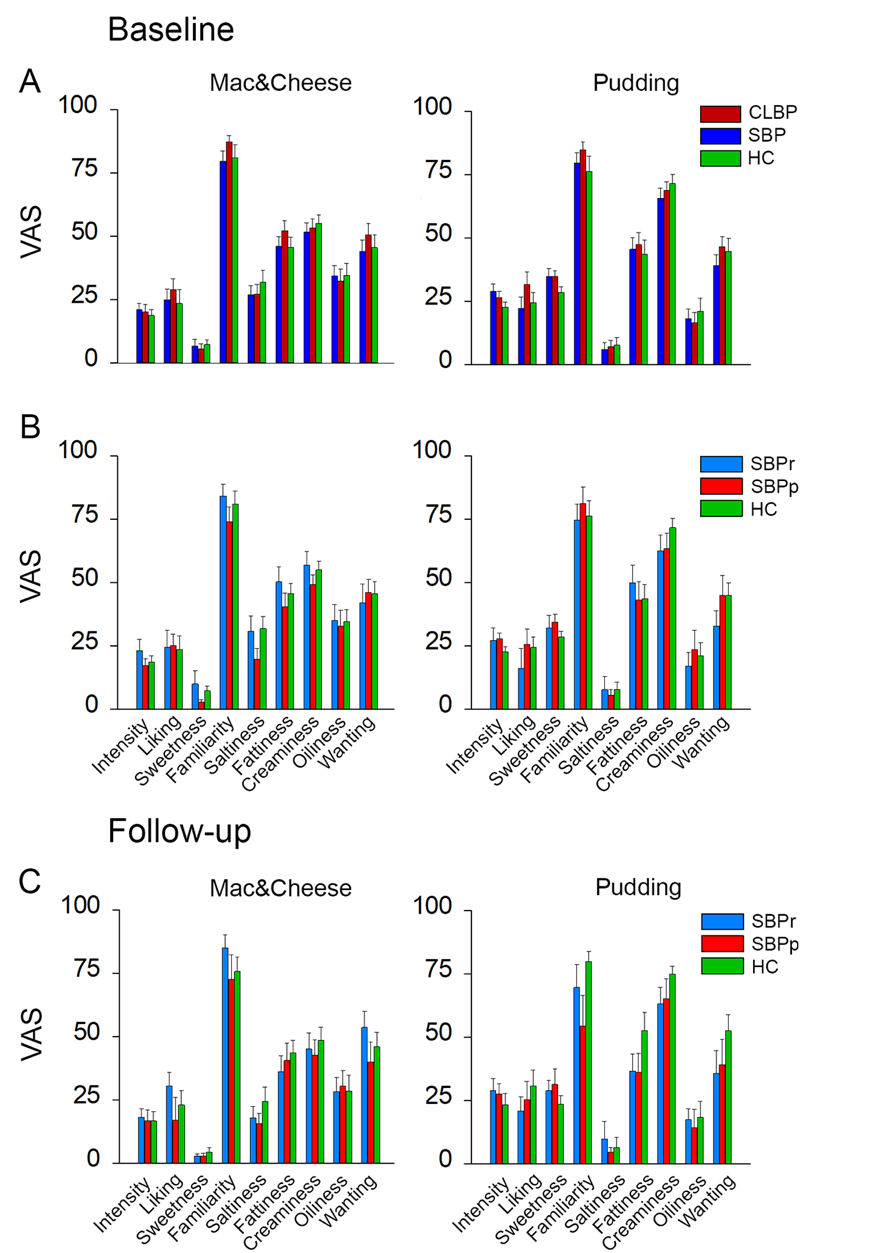


**S6 Fig**. Psychological ratings during session 2 at baseline and follow-up. (**A**) Ratings of MacCheese (left) and pudding (right) for SBP, CLBP and HC participants at baseline; (**B**) Ratings of MacCheese (left) and pudding (right) for SBPr, SBPp and HC participants at baseline; (**C**) Ratings of MacCheese (left) and pudding (right) for SBPr, SBPp and HC at follow-up. CLBP, chronic low back pain; SBP, subacute back pain; SBPr, subacute back pain recovered; SBPp, subacute back pain persistent; HC, Healthy Control. No significant differences were found among groups.
