# Supplementary material for: Chronic pain precedes disrupted eating behavior in low-back pain patients
Source: PLoS One. 2022 Feb 10;17(2):e0263527. doi: 10.1371/journal.pone.0263527 (PMC8830732; doi:10.1371/journal.pone.0263527)
Supplement: S7 Fig — No changes in any of the groups were found from baseline to 1-year follow-up. SBPr, subacute back pain recovered; SBPp, subacute back pain persistent; HC, Healthy Control. (DOCX) [file pone.0263527.s007.docx]

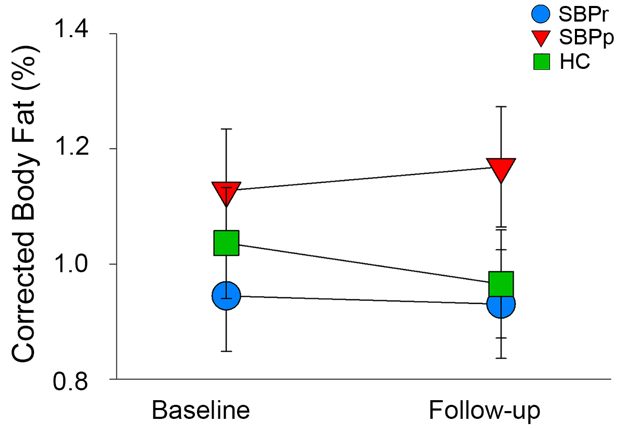


**S7 Fig**. Body fat change from baseline to follow-up. No changes in any of the groups were found from baseline to 1-year follow-up. SBPr, subacute back pain recovered; SBPp, subacute back pain persistent; HC, Healthy Control.
