# Supplementary material for: Chronic pain precedes disrupted eating behavior in low-back pain patients
Source: PLoS One. 2022 Feb 10;17(2):e0263527. doi: 10.1371/journal.pone.0263527 (PMC8830732; doi:10.1371/journal.pone.0263527)
Supplement: S1 Table — SBP (42), CLBP (37), HC (29). a Values are expressed as mean ± SEM. b Calculated by repeated measure ANOVA. (DOCX) [file pone.0263527.s008.docx]

**S1 Table**. Internal state ratings for SBP and CLBP patients, and for healthy subjects for session 1 at baseline ^a^

|  |  | SBP | CLBP | HC | Group Effect | Time Effect | Group x Time ^b^ |
| --- | --- | --- | --- | --- | --- | --- | --- |
| hunger | pre | 20.1 ± 3.3 | 28.5 ± 3.5 | 27.4 ± 3.9 | 0.137 | 0.006^*^ | 0.949 |
|  | post | 24.0 ± 3.7 | 33.5 ± 4.0 | 32.1 ± 4.5 |  |  |  |
| fullness | pre | 24.8 ± 3.3 | 19.9 ± 3.6 | 32.5 ± 4.0 | 0.192 | 0.770 | 0.211 |
|  | post | 23.3 ± 3.6 | 23.2 ± 3.9 | 29.4 ± 4.4 |  |  |  |
| Thirst | pre | 21.5 ± 3.6 | 29.0 ± 3.9 | 30.3 ± 4.3 | 0.142 | 0.740 | 0.811 |
|  | post | 19.9 ± 3.9 | 29.9 ± 4.1 | 29.2 ± 4.7 |  |  |  |

SBP (42), CLBP (37), HC (29).

a Values are expressed as mean ± SEM.

b Calculated by repeated measure ANOVA.
