# Supplementary material for: Chronic pain precedes disrupted eating behavior in low-back pain patients
Source: PLoS One. 2022 Feb 10;17(2):e0263527. doi: 10.1371/journal.pone.0263527 (PMC8830732; doi:10.1371/journal.pone.0263527)
Supplement: S2 Table — a Values are expressed as mean ± SEM. b Calculated by repeated measure ANOVA. (DOCX) [file pone.0263527.s009.docx]

**S2 Table**. Internal state ratings for SBPr and SBPp patients, and for healthy subjects for session 1 at baseline ^a^

|  |  | SBPr | SBPp | HC | Group Effect | Time Effect | Group x Time ^b^ |
| --- | --- | --- | --- | --- | --- | --- | --- |
| hunger | pre | 21.2 ± 5.1 | 21.6 ± 5.3 | 27.4 ± 3.9 | 0.476 | 0.073 | 0.963 |
|  | post | 25.1 ± 5.8 | 25.0 ± 6.0 | 32.1 ± 4.4 |  |  |  |
| fullness | pre | 30.6 ± 5.6 | 26.2 ± 5.8 | 32.5 ± 4.3 | 0.804 | 0.470 | 0.749 |
|  | post | 29.0 ± 5.7 | 26.6 ± 5.9 | 29.4 ± 4.4 |  |  |  |
| Thirst | pre | 24.0 ± 5.6 | 24.9 ± 5.8 | 30.3 ± 4.3 | 0.545 | 0.422 | 0.941 |
|  | post | 21.4 ± 5.9 | 23.7 ± 6.1 | 29.2 ± 4.6 |  |  |  |

a Values are expressed as mean ± SEM.

b Calculated by repeated measure ANOVA.
