# Supplementary material for: Chronic pain precedes disrupted eating behavior in low-back pain patients
Source: PLoS One. 2022 Feb 10;17(2):e0263527. doi: 10.1371/journal.pone.0263527 (PMC8830732; doi:10.1371/journal.pone.0263527)
Supplement: S3 Table — a Values are expressed as mean ± SEM. b Calculated by repeated measure ANOVA. (DOCX) [file pone.0263527.s010.docx]

**S3 Table**. Internal state ratings for SBPr and SBPp patients, and for healthy subjects for session 1 at follow-up ^a^

|  |  | SBPr | SBPp | HC | Group Effect | Time Effect | Group x Time ^b^ |
| --- | --- | --- | --- | --- | --- | --- | --- |
| hunger | pre | 8.7 ± 5.2 | 29.7 ± 5.4 | 9.9 ± 4.9 | 0.133 | 0.006^*^ | 0.067 |
|  | post | 23.5 ± 6.5 | 28.6 ± 6.8 | 19.2 ± 6.1 |  |  |  |
| fullness | pre | 21.6 ± 6.7 | 16.7 ± 7.0 | 25.7 ± 6.3 | 0.757 | 0.340 | 0.639 |
|  | post | 21.9 ± 7.0 | 23.2 ± 7.3 | 27.0 ± 6.5 |  |  |  |
| Thirst | pre | 17.0 ± 5.8 | 26.1 ± 6.0 | 13.3 ± 5.4 | 0.362 | 0.240 | 0.619 |
|  | post | 17.1 ± 6.9 | 28.4 ± 7.2 | 18.2 ± 6.4 |  |  |  |

a Values are expressed as mean ± SEM.

b Calculated by repeated measure ANOVA.
