# Supplementary material for: Chronic pain precedes disrupted eating behavior in low-back pain patients
Source: PLoS One. 2022 Feb 10;17(2):e0263527. doi: 10.1371/journal.pone.0263527 (PMC8830732; doi:10.1371/journal.pone.0263527)
Supplement: S4 Table — a F Values are results of a mixed 2-way ANOVA where group (SBP vs CLBP vs healthy) was a factor and stimulus concentration the repeated measure. * p < .05, ** p < .005, *** p < .001. (DOCX) [file pone.0263527.s011.docx]

**S4 Table.** Comparison of SBP, CLBP patients’ and healthy subjects’ ratings of puddings and jello during session 1 ^a^

|  | Group, F_2,127_ | Stimulus concentration, F_3,381_ | Group x Concentration, F_6,381_ |
| --- | --- | --- | --- |
| Pudding |  |  |  |
| Liking | 1.180 | 4.734^**^ | 2.200^*^ |
| Intensity | 0.798 | 0.445 | 1.539 |
| Sweetness | 2.004 | 0.938 | 0.528 |
| Familiarity | 0.078 | 0.600 | 0.909 |
| Fattiness | 0.064 | 2.676^*^ | 1.418 |
| Creaminess | 0.200 | 10.180^*^ | 0.840 |
| Oiliness | 1.126 | 1.960 | 1.407 |
| Wanting | 0.643 | 4.305^*^ | 0.736 |
| Jello |  |  |  |
| Liking | 0.100 | 166.100^***^ | 0.900 |
| Intensity | 3.160^*^ | 22.150^***^ | 0.830 |
| Sweetness | 3.100 | 209.900^***^ | 2.200 |
| Familiarity | 0.600 | 125.900^***^ | 0.909 |
| Fattiness | 2.170 | 19.070^***^ | 2.300 |
| Creaminess | 0.650 | 15.130^***^ | 0.670 |
| Oiliness | 0.467 | 1.955 | 2.511 |
| Wanting | 1.200 | 128.000^***^ | 1.200 |
| a F Values are results of a mixed 2-way ANOVA where group (SBP vs CLBP vs healthy) was a factor and stimulus concentration the repeated measure. | | | |
| ^*^ p < .05, ^**^ p < .005, ^***^ p < .001. | | | |
