# Supplementary material for: Chronic pain precedes disrupted eating behavior in low-back pain patients
Source: PLoS One. 2022 Feb 10;17(2):e0263527. doi: 10.1371/journal.pone.0263527 (PMC8830732; doi:10.1371/journal.pone.0263527)
Supplement: S5 Table — a F Values are results of a mixed 2-way ANOVA where group (SBPr vs SBPp vs healthy) was a factor and stimulus concentration the repeated measure. * p < .05, ** p < .005, *** p < .001. (DOCX) [file pone.0263527.s012.docx]

**S5 Table.** Comparison of SBPr, SBPp patients’ and healthy subjects’ ratings of puddings and jello during session 1 baseline ^a^

|  | Group, F_2,69_ | Stimulus concentration, F_3,207_ | Group x Concentration, F_6,207_ |
| --- | --- | --- | --- |
| Pudding |  |  |  |
| Liking | 1.752 | 0.388 | 3.220^*^ |
| Intensity | 0.120 | 0.342 | 0.441 |
| Sweetness | 0.298 | 0.231 | 0.530 |
| Familiarity | 1.031 | 0.443 | 1.076 |
| Fattiness | 0.954 | 1.872 | 0.792 |
| Creaminess | 0.076 | 6.847^***^ | 1.027 |
| Oiliness | 0.293 | 3.039 | 0.148 |
| Wanting | 0.495 | 0.447 | 1.401 |
| Jello |  |  |  |
| Liking | 0.120 | 77.840^***^ | 0.680 |
| Intensity | 0.350 | 14.710^***^ | 0.530 |
| Sweetness | 0.020 | 85.320^***^ | 0.470 |
| Familiarity | 2.360 | 59.600^***^ | 1.010 |
| Fattiness | 2.039 | 7.060^***^ | 2.274 |
| Creaminess | 2.357 | 8.474^***^ | 0.641 |
| Oiliness | 0.841 | 2.375 | 2.585^*^ |
| Wanting | 0.100 | 66.540^***^ | 0.780 |
| a F Values are results of a mixed 2-way ANOVA where group (SBPr vs SBPp vs healthy) was a factor and stimulus concentration the repeated measure. | | | |
| ^*^ p < .05, ^**^ p < .005, ^***^ p < .001. | | | |
