# Supplementary material for: Chronic pain precedes disrupted eating behavior in low-back pain patients
Source: PLoS One. 2022 Feb 10;17(2):e0263527. doi: 10.1371/journal.pone.0263527 (PMC8830732; doi:10.1371/journal.pone.0263527)
Supplement: S6 Table — a F Values are results of a mixed 2-way ANOVA where group (SBPr vs SBPp vs healthy) was a factor and stimulus concentration the repeated measure. * p < .05, ** p < .005, *** p < .001. (DOCX) [file pone.0263527.s013.docx]

**S6 Table.** Comparison of SBPr, SBPp patients’ and healthy subjects’ ratings of puddings and jello during session 1 follow-up ^a^

|  | Group, F_2,42_ | Stimulus concentration, F_3,126_ | Group x Concentration, F_6,126_ |
| --- | --- | --- | --- |
| Pudding |  |  |  |
| Liking | 3.269^*^ | 2.509 | 0.616 |
| Intensity | 0.455 | 0.284 | 1.419 |
| Sweetness | 0.042 | 1.614 | 1.034 |
| Familiarity | 4.358^*^ | 0.036 | 0.882 |
| Fattiness | 0.509 | 0.799 | 0.761 |
| Creaminess | 0.708 | 4.013^**^ | 0.468 |
| Oiliness | 0.245 | 2.182 | 0.774 |
| Wanting | 3.101 | 1.690 | 0.548 |
| Jello |  |  |  |
| Liking | 1.390 | 49.100^***^ | 1.590 |
| Intensity | 1.142 | 2.889^*^ | 0.482 |
| Sweetness | 0.630 | 57.710^***^ | 0.260 |
| Familiarity | 3.800^*^ | 54.450^***^ | 3.230^*^ |
| Fattiness | 4.229^*^ | 3.118^*^ | 2.120 |
| Creaminess | 2.394 | 4.286^*^ | 1.424 |
| Oiliness | 0.501 | 0.539 | 0.708 |
| Wanting | 4.280^*^ | 46.860^***^ | 2.200^*^ |
| a F Values are results of a mixed 2-way ANOVA where group (SBPr vs SBPp vs healthy) was a factor and stimulus concentration the repeated measure.  ^*^ p < .05, ^**^ p < .005, ^***^ p < .001. | | | |
|  | |  |  |
