# Supplementary material for: Chronic pain precedes disrupted eating behavior in low-back pain patients
Source: PLoS One. 2022 Feb 10;17(2):e0263527. doi: 10.1371/journal.pone.0263527 (PMC8830732; doi:10.1371/journal.pone.0263527)
Supplement: S7 Table — a Values are expressed as mean ± SEM. b Calculated by repeated measure ANOVA. (DOCX) [file pone.0263527.s014.docx]

**S7 Table**. Internal state ratings for session 2 at baseline and follow-up ^a^

|  |  | SBP | CLBP | HC | Group Effect | Time Effect | | | Group x Time ^b^ | |
| --- | --- | --- | --- | --- | --- | --- | --- | --- | --- | --- |
| hunger | pre | 59.3 ± 3.5 | 59.4 ± 3.9 | 58.9 ± 3.8 | 0.419 | 0.000^*^ | | | 0.195 | |
|  | post | 26.9 ± 3.8 | 26.8 ± 4.0 | 26.7 ± 4.1 |  |  | | |  | |
| fullness | pre | 14.1 ± 3.0 | 10.9 ± 2.9 | 11.4 ± 3.2 | 0.646 | 0.000^*^ | | | 0.270 | |
|  | post | 42.1 ± 4.5 | 49.0 ± 4.8 | 47.8 ± 4.8 |  |  | | |  | |
| Thirst | pre | 43.8 ± 4.1 | 45.3 ± 4.5 | 44.5 ± 4.4 | 0.861 | 0.930 | | | 0.386 | |
|  | post | 48.4 ± 4.1 | 43.7 ± 4.5 | 42.0 ± 4.4 |  |  | | |  | |
| **Baseline** | | | | | | | | | | |
|  |  | SBPr | SBPp | HC | Group Effect | | | Time Effect | Group x Time ^b^ | |
| hunger | pre | 56.1 ± 5.2 | 62.2 ± 6.3 | 69.0 ± 4.3 | 0.513 | 0.000^*^ | | | 0.043^*^ | |
|  | post | 31.5 ± 5.3 | 18.1 ± 6.3 | 25.2 ± 4.3 |  |  | | |  | |
| fullness | pre | 10.6 ± 4.5 | 23.0 ± 5.4 | 9.7 ± 3.7 | 0.263 | 0.000^*^ | | | 0.204 | |
|  | post | 41.6 ± 6.4 | 44.0 ± 7.6 | 48.2 ± 5.2 |  |  | | |  | |
| Thirst | pre | 49.3 ± 5.9 | 35.5 ± 7.1 | 49.1 ± 4.8 | 0.858 | 0.608 | | | 0.027^*^ | |
|  | post | 43.8 ± 5.6 | 51.9 ± 6.7 | 45.3 ± 4.6 |  |  | | |  | |
| **Follow-up** | | | | | | | | | | |
|  |  | SBPr | SBPp | HC | Group Effect | | Time Effect | | | Group x Time ^b^ |
| hunger | pre | 49.4 ± 5.2 | 53.5 ± 6.2 | 61.4 ± 5.0 | 0.262 | | 0.000^*^ | | | 0.731 |
|  | post | 16.9 ± 5.0 | 15.8 ± 6.3 | 22.6 ± 4.8 |  | |  | | |  |
| fullness | pre | 10.7 ± 4.1 | 7.6 ± 6.0 | 12.6 ± 3.9 | 0.271 | | 0.000^*^ | | | 0.488 |
|  | post | 43.1 ± 7.5 | 31.4 ± 8.5 | 49.5 ± 7.2 |  | |  | | |  |
| Thirst | pre | 32.7 ± 6.0 | 38.3 ± 7.3 | 41.6 ± 5.8 | 0.757 | | 0.529 | | | 0.752 |
|  | post | 37.4 ± 7.5 | 42.1 ± 8.7 | 40.2 ± 7.3 |  | |  | | |  |
| a Values are expressed as mean ± SEM.  b Calculated by repeated measure ANOVA | | | | | | | | | | |
