# Supplementary material for: Chronic pain precedes disrupted eating behavior in low-back pain patients
Source: PLoS One. 2022 Feb 10;17(2):e0263527. doi: 10.1371/journal.pone.0263527 (PMC8830732; doi:10.1371/journal.pone.0263527)
Supplement: S9 Table — a Values are expressed as mean ± SEM. b Results of a one-way ANOVA among groups (SBP vs CLBP vs healthy). (DOCX) [file pone.0263527.s016.docx]

**S9 Table.** Consumed food attribute ratings for SBPr, SBPp patients and healthy subjects for session 2 at baseline ^a^

| Parameter | SBP | | CLBP | | HC | | | p-value ^b^ |  |
| --- | --- | --- | --- | --- | --- | --- | --- | --- | --- |
| *Mac & Cheese* | |  | |  | |  |  | | |
| Intensity | 21.12 ± 2.49 | | 20.44 ± 2.77 | | 18.77 ± 2.38 | | | 0.82 |  |
| Liking | 24.93 ± 4.40 | | 28.83 ± 4.90 | | 23.58 ± 5.37 | | | 0.74 |  |
| Sweetness | 6.79 ± 2.63 | | 5.99 ± 2.38 | | 7.30 ± 1.87 | | | 0.93 |  |
| Familiarity | 79.77 ± 3.96 | | 88.18 ± 4.00 | | 81.04 ± 5.04 | | | 0.26 |  |
| Fattiness | 46.17 ± 3.83 | | 52.42 ± 4.12 | | 45.67 ± 3.98 | | | 0.43 |  |
| Creaminess | 51.77 ± 3.57 | | 54.03 ± 3.71 | | 55.03 ± 3.40 | | | 0.80 |  |
| Oiliness | 34.40 ± 4.05 | | 32.35 ± 4.66 | | 34.60 ± 4.78 | | | 0.93 |  |
| Wanting | 44.12 ± 4.43 | | 50.69 ± 4.87 | | 45.59 ± 4.92 | | | 0.59 |  |
| *Pudding* |  | |  | |  | | |  |  |
| Intensity | 28.95 ± 2.84 | | 26.35 ± 2.69 | | 22.57 ± 2.02 | | | 0.23 |  |
| Liking | 22.13 ± 4.61 | | 31.09 ± 4.84 | | 24.35 ± 4.14 | | | 0.37 |  |
| Sweetness | 34.81 ± 3.02 | | 35.24 ± 2.73 | | 28.58 ± 2.08 | | | 0.16 |  |
| Familiarity | 79.54 ± 4.04 | | 85.25 ± 4.47 | | 76.30 ± 5.98 | | | 0.39 |  |
| Fattiness | 45.56 ± 4.55 | | 47.40 ± 5.18 | | 43.64 ± 5.53 | | | 0.88 |  |
| Creaminess | 65.63 ± 4.07 | | 69.48 ± 3.96 | | 71.61 ± 3.57 | | | 0.53 |  |
| Oiliness | 18.00 ± 3.98 | | 17.38 ± 4.55 | | 21.09 ± 5.13 | | | 0.83 |  |
| Wanting | 39.01 ± 4.45 | | 46.07 ± 4.68 | | 44.79 ± 4.95 | | | 0.48 |  |
| a Values are expressed as mean ± SEM.  b Results of a one-way ANOVA among groups (SBP vs CLBP vs healthy). | | | | | | | | |  |
