# Supplementary material for: Chronic pain precedes disrupted eating behavior in low-back pain patients
Source: PLoS One. 2022 Feb 10;17(2):e0263527. doi: 10.1371/journal.pone.0263527 (PMC8830732; doi:10.1371/journal.pone.0263527)
Supplement: S10 Table — a Values are expressed as mean ± SEM. b Results of a one-way ANOVA among groups (SBP vs CLBP vs healthy). (DOCX) [file pone.0263527.s017.docx]

**S10 Table**. Ratings for SBPr, SBPp and HC groups at session 2 at follow-up

|  | SBPr | SBPp | HC | p-value ^b^ |
| --- | --- | --- | --- | --- |
| *Mac &Cheese* | |  |  |  |
| Intensity | 18.29 ± 3.33 | 16.90 ± 4.13 | 16.74 ± 3.62 | 0.88 |
| Liking | 30.52 ± 5.43 | 17.01 ± 8.94 | 23.15 ± 5.51 | 0.74 |
| Sweetness | 2.83 ± 0.90 | 2.89 ± 0.97 | 4.39 ± 1.94 | 0.47 |
| Familiarity | 85.08 ± 5.10 | 72.78 ± 9.59 | 75.78 ± 5.72 | 0.68 |
| Fattiness | 36.20 ± 6.38 | 40.62 ± 6.89 | 43.61 ± 4.99 | 0.94 |
| Creaminess | 45.13 ± 6.28 | 42.74 ± 6.02 | 48.58 ± 5.19 | 0.52 |
| Oiliness | 28.42 ± 5.65 | 30.52 ± 6.22 | 28.56 ± 6.24 | 0.98 |
| Wanting | 53.71 ± 6.45 | 39.93 ± 7.86 | 46.00 ± 5.83 | 0.53 |
| *Pudding* |  |  |  |  |
| Intensity | 28.95 ± 4.72 | 27.55 ± 3.99 | 23.33 ± 4.40 | 0.50 |
| Liking | 20.83 ± 5.55 | 25.22 ± 7.33 | 30.75 ± 6.30 | 0.14 |
| Sweetness | 28.86 ± 4.12 | 31.30 ± 6.07 | 23.46 ± 3.34 | 0.37 |
| Familiarity | 69.66 ± 9.12 | 54.32 ± 12.23 | 79.88 ± 3.92 | 0.73 |
| Fattiness | 36.53 ± 6.90 | 36.12 ± 7.38 | 52.60 ± 7.06 | 0.76 |
| Creaminess | 63.14 ± 6.62 | 65.23 ± 7.86 | 74.83 ± 3.13 | 0.19 |
| Oiliness | 17.50 ± 4.22 | 14.35 ± 7.06 | 18.38 ± 6.27 | 0.87 |
| Wanting | 35.70 ± 8.88 | 39.02 ± 10.08 | 52.48 ± 6.45 | 0.09 |
| a Values are expressed as mean ± SEM.  b Results of a one-way ANOVA among groups (SBP vs CLBP vs healthy). | | | | |
