# Supplementary material for: Chronic pain precedes disrupted eating behavior in low-back pain patients
Source: PLoS One. 2022 Feb 10;17(2):e0263527. doi: 10.1371/journal.pone.0263527 (PMC8830732; doi:10.1371/journal.pone.0263527)
Supplement: Attachment — Submitted filename: Response2Rev.docx [file pone.0263527.s019.docx]

We would like to thank both reviewers for their interest in our work and their constructive feedback which helped improve the manuscript. Please find below point by point response to the reviewers comments.

*Reviewer #1: This is a well done study addressing a complex relationship between the hedonic qualities of food and chronic pain along with evaluation of the NAc. The findings are in many ways counter-intuitive and somewhat difficult to appreciate but have important implications.*

Thank you!

*The main suggestion is that the abstract should be re-written to provide the interpretation of the findings regarding the role of the NAc in disruption of hedonic quality in SBPr patients while a dysfunctional accumbens in the SBPp group protects them against disruption.*

As per the reviewer’s suggestion we re-wrote the abstract to provide the interpretation of the role of the NAc in the disruption of hedonic processing. We now write:

Chronic pain is associated with anhedonia and decreased motivation. These behavioral alterations have been linked to alterations in the limbic brain and could explain the increased risk for obesity in pain patients. The mechanism of these behavioral changes and how they set in in relation to the development of chronic pain remain however poorly understood. Here we asked how eating behavior is affected in low-back pain patients before and after they transition to chronic pain, compared to patients whose pain subsides. Additionally, we assessed how the hedonic perception of fat-rich food, which is altered in chronic pain patients, relates to the properties of the nucleus accumbens in this patients’ population. We hypothesized that the accumbens will be directly implicated in the hedonic processing of fat-rich food in pain patients because of its well-established role in hedonic feeding and fat ingestion, and its emerging role in chronic pain. Accordingly, we used behavioral assays and structural brain imaging to test sub-acute back pain patients (SBP) and healthy control subjects at baseline and at approximately one-year follow-up. We also studied a sample of chronic low-back pain patients (CLBP) at one time point only. We found that SBP patients who recovered at follow-up (SBPr) and CLBP patients showed disrupted eating behaviors. In contrast, SBP patients who persisted in having pain at follow-up (SBPp) showed intact eating behavior. From a neurological standpoint, only SBPp and CLBP patients showed a strong and direct relationship between hedonic perception of fat-rich food and nucleus accumbens volume suggesting that accumbens alterations observed in SBPp patients in previous works might protect them from hedonic eating disruptions during the early course of the illness. We conclude that disrupted eating behavior specifically sets in after pain chronification and is accompanied by structural changes in the nucleus accumbens.

*It would also be helpful to provide better definition in many places in the manuscript of the term "disruption" - what exactly was being disrupted in the context of the sentence?*

We thank the reviewer for this suggestion. We tried to rephrase or explain the word disruption to be more specific all through the manuscript in the revised version.

*It will also be interesting to see the outcomes of the proposed opioid studies.*

We strongly agree with the reviewer on this future step. We are in the process of applying for funding to do these experiments.

*Reviewer #2: Yezhe Lin and collaborators have investigated whether Chronic pain precedes disrupted eating behavior in low-back pain patients
This research is original and suggest that chronic pain precedes disrupted eating behavior in low-back pain patients and that such disruption is directly related to structural changes in nucleus accumbens. In spite of that, some aspects need to be clarified.
Globaly , The manuscript is difficult to follow and deserves to be simplified or reorganized by example:*

*- In Methods section, I suggest to include a schematic diagram describing the groups and procedures*

We thank the reviewer for this excellent suggestion. We now added a new figure (Figure 1) which depicts a schematic diagram describing the groups and the procedures.

*- Section results should be simplified and the figures numbers must follow as far as possible their order of quotation in the manuscript*

Per the reviewer’s suggestion we have now simplified the presentation of the results. We added a table (Table 4) to report the caloric intake separately from tables 1-3. We as much as possible now quote the figures in the order of their numbers. In addition we grouped supplementary tables 7-9 into one supplementary table 7 hence reducing the number of tables in the supporting information from 12 to 10.

- In all the figures , bar graph shall be a contrasting colour.

*Per the reviewer’s suggestion we changed all the colors of all our figures to ensure an adequate contrast.*

- Legend of figure 2 (A-C) is omitted

The legend of Figure 2 (now Figure 3) were inserted.

*- Insert the groups in fig 2 and Fig 5*

We inserted the groups in Figures 2-5 which are now numbered Figure 3-6 because we added a new Figure 1.

*- A few tapping errors must be corrected*

Typing errors were corrected.
